# Genomics Analysis of *Bacillus megaterium* 1259 as a Probiotic and Its Effects on Performance in Lactating Dairy Cows

**DOI:** 10.3390/ani11020397

**Published:** 2021-02-04

**Authors:** Bobo Deng, Lin Wang, Qianbo Ma, Tongshui Yu, Dalin Liu, Yi Dai, Guoqi Zhao

**Affiliations:** 1 College of Animal Science and Technology, Yangzhou University, Yangzhou 225009, China; 18168960899@163.com (B.D.); mqb1601555436@163.com (Q.M.); 15205518823@139.com (T.Y.); liudalin2020@163.com (D.L.); cminghui93@gmail.com (Y.D.); gqzhao@yzu.edu.cn (G.Z.); 2 Institutes of Agricultural Science and Technology Development, Yangzhou University, Yangzhou 225009, China; 3 Joint International Research Laboratory of Agriculture and Agri-Product Safety, The Ministry of Education of China, Yangzhou University, Yangzhou 225009, China

**Keywords:** *Bacillus megaterium* 1259, complete genome sequences, milk production, ruminal fermentation, blood metabolites, nitrogen utilization

## Abstract

**Simple Summary:**

Probiotics play a vital role in animal production. *Bacillus megaterium* 1259, as a novel bacterium, has been used as a probiotic in poultry feed. In this study, we analyzed the genome sequence and genetic characteristics of *Bacillus megaterium* 1259 in order to have a deep understanding of its genomics information. Moreover, the effects of *Bacillus megaterium* 1259 as a probiotic on lactating dairy cow performance were determined due to the different digestive systems of dairy cows from poultry. The results suggest that *Bacillus megaterium* 1259 can increase milk production and will not bring any side effects on blood metabolites in dairy cows.

**Abstract:**

In this study, we isolated a novel bacterium, *Bacillus megaterium* 1259 (BM1259), from chicken manure. Whole-genome sequencing analysis showed that the BM1259 complete genome is composed of a 5,043,095 bp circular chromosome and three circular plasmids, and it encodes 5379 coding genes and 182 RNA genes. Among these genes, a series of nitrate assimilation-related genes and pathways were identified, implying a potential role of BM1259 in nitrate metabolism. In addition, 24 lactating Holstein dairy cows were randomly assigned to four groups that were fed a total mixed ration (TMR) diet only (C), a TMR diet supplemented with 5 g/day of BM1259 (T1), a TMR diet supplemented with 10 g/day of BM1259 (T2), or a TMR diet supplemented with 15 g/day of BM1259 (T3). The results showed that supplementing dairy cows with 15 g/day of BM1259 increased 4% fat-corrected milk production. The molar proportion of propionate (C3) was significantly higher in T2 than in C. The C2:C3 ratio of T3 was higher than those of C and T2. No negative effect of BM1259 on blood indicators was detected. This study demonstrates BM1259 can be applied as a potential probiotic to improve nitrogen utilization and milk production in lactating dairy cows.

## 1. Introduction

Probiotics are live microorganisms that bring health benefits to the host when administered in adequate amounts [1]. They play a vital role in animal production due to their non-residual and non-polluting nature. Previous studies have reported the effects of different probiotics in animal diets, such as enhancing the growth rate, increasing feed intake and feed efficiency, improving carcass yield and quality, increasing nutrient digestibility, and controlling enteric pathogens [2,3,4,5,6]. *Bacillus*, spore-forming bacteria, are becoming increasingly popular as probiotics used in animal feed. For example, *Bacillus* spp. strains were analyzed for usage as probiotic additives in pig feed [7]. Kritas et al. demonstrated that supplementing ewe feed with *Bacillus* sp. probiotics may promote subsequent milk yields and fat and protein contents [8].

Studying the effects of a certain microbial strain on various animal species, growth conditions, and diet types may help to clarify the conditions under which probiotics could work in animal production. In dairy cow production, *Bacillus subtilis natto*, as a probiotic, has positive effects on rumen fermentation, the ruminal microbiome, and milk production in dairy cows [9]. Another *Bacillus* sp., *Bacillus licheniformis*, improved ruminal apparent nutrient digestibility of organic matter, neutral detergent fiber, and acid detergent fiber in Holstein cows [10].

*Bacillus megaterium* is a gram-positive, aerobic spore-forming neutralophilic bacterium that is ubiquitous in various environments but commonly survives in the soil. It has a potential role as an industrial probiotic due to its adaptability to a wide temperature environment (3–45 °C) and utilization of different carbon sources [11,12]. To date, most studies have focused on its beneficial effects on plants, such as biocontrol ability against plant pathogens [13,14]. Although another series of studies suggests that *B. megaterium* has the potential to be applied as a probiotic in the feed industry [15,16], further study on the effect and safety of probiotics, especially novel probiotics, is required. *B. megaterium* 1259 (BM1259) was isolated from chicken manure, and fermentation technology for producing it has been well developed. In our previous studies, we found that diets supplemented with BM1259 could improve the performance of laying hens and decrease the ammonia nitrogen emissions of excrement [17]. To date, the genetic mechanisms of BM1259 are not fully understood. In addition, the digestive system in ruminants is different from that in chickens due to the rumen. Hence, the effect of BM1259 needs to be investigated before use in ruminants.

Given the dearth of genetic information, we analyzed the genome sequence and genetic characteristics of BM1259, which might be used as a new feed additive for animal production. The second objective of this study was to investigate the effects of BM1259 on lactating dairy cow performance. This work will provide genomic information for a deep understanding of BM1259 and provide a new bacterium strain for the probiotic industry for dairy cow production.

## 2. Materials and Methods

### 2.1. Bacterial Isolate

**Figure 1:**
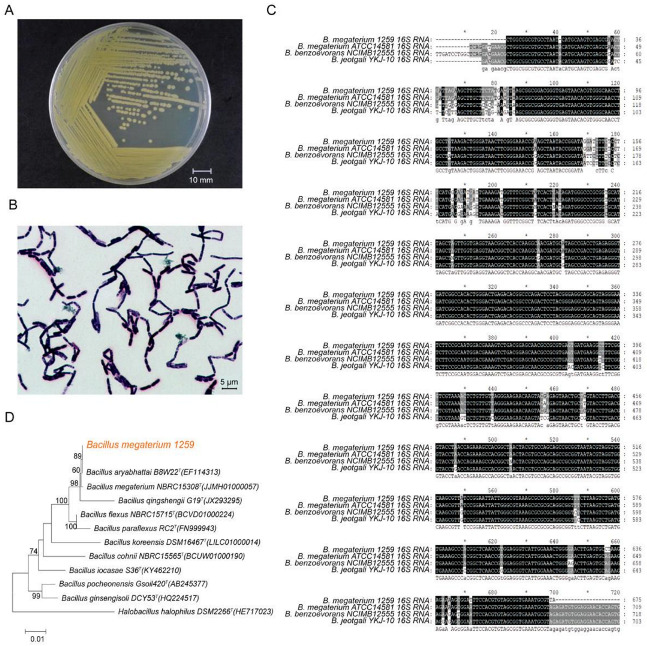
Identification of BM1259. (**A**) Culture and (**B**) the morphological characteristics of BM1259. The scale bar corresponds to 10 mm and 5 µm. (**C**) Multiple alignment of 16S rRNA sequences from BM1259, *Bacillus megaterium ATCC 14581*, *Bacillus benzoevorans NCIMB 12555,* and *Bacillus jeotaglli YKJ-10*. Conserved residues are shown in an inverse rendition, and residues that are not identical but at least similar to the column consensus are shown in a gray background rendition. (**D**) Phylogenetic tree of the 16S rRNA sequence from BM1259, *Bacillus megaterium NBRC 15308T* (JJMH01000057), *Bacillus qingshengii G19T* (JX293295), *Bacillus flexus NBRC 15715T* (BCVD01000224), *Bacillus koreensis DSM 16467T* (LILC01000014), *Bacillus cohnii NBRC 15565T* (BCUW01000190), *Bacillus iocasae S36T* (KY462210), *Bacillus pocheonensis Gsoil 420T* (AB245377), and *Bacillus ginsengisoli DCY53T* (HQ224517); *Halobacillus halophilus DSM 2266T* (HE717023) was included as an outgroup.

The bacterial strain BM1259 was isolated from chicken manure in Yangzhou city and deposited in the China General Microbiological Culture Collection Center (CGMCC No 1259). This strain was identified based on the 16S ribosomal DNA sequence by multiple alignment and phylogenetic analysis and cultured in our own laboratory (Figure 1A) in this study.

### 2.2. Genome Sequencing, Assembly, and Annotation

The genome sequence data were deposited in NCBI GenBank, and the accession number is PRJNA506612. The complete genomic DNA of BM1259 was obtained by a QIAGEN Genomic DNA extraction kit (QIAGEN, Dusseldorf, Germany), according to the standard manufacturer methods, and quantified using a NanoDrop One UV-Vis spectrophotometer (Thermo Fisher Scientific, Waltham, USA). A nanopore sequencing library of genomic DNA was prepared at Nanjing Aurora Gene Technology Co., Ltd., China. All long DNA fragments from extracted DNA samples were selected using the BluePippin system (Sage Science, Beverly, USA). The ends of all long DNA fragments were attached with adapters supplied by the Ligation Sequencing kit (SQK-LSK109, Oxford, UK). A Qubit 3.0 Fluorometer (Thermo Fisher Scientific, Waltham, USA) was used to evaluate the size of library fragments. Finally, the DNA library was added into a flow cell, and sequencing was performed on the GridION sequencing platform. The raw signal data were translated into raw DNA reads using base calling. Overlap–layout–consensus algorithms were used for the assembly of nanopore sequencing reads to generate an overlap graph. Then, a draft assembly was constructed according to previous methods [18]. The assembled genome was drawn by the Circos 1.7.11 (http://circos.ca) [19]. Based on assembled genomic sequences, coding sequences (CDSs), tRNA, rRNA, ncRNA, clustered regularly interspaced short palindromic repeat sequences (CRISPRs), and genomic islands were predicted using Prodigal, tRNAscan-SE, RNAmmer, infernal, minced, and Islander, respectively. All CDSs were annotated based on the Gene Ontology (GO), Kyoto Encyclopedia of Genes and Genomes (KEGG), Cluster of Orthologous Groups of proteins (COG), Reference sequences (Refseq), Pfam, and TIGRFAM databases.

### 2.3. Multiple Alignments and Phylogenetic Analyses of 16S rRNA Sequences

Based on the 16S rRNA gene from the BM1259 genome, the full-length 16S rRNA sequence was amplified using PrimeSTAR Max DNA Polymerase kits (Takara, Otsu, Japan). Sequences encoding 16S rRNA of BM1259 and other bacteria were initially aligned with Clustal W and displayed using GeneDoc software. Phylogenetic trees were analyzed using the maximum likelihood method in MEGA 6 (http://www.megasoftware.net/), as described in a previous report [20].

### 2.4. Animal Diet, and Experimental Design

An animal experiment was conducted at the Kangyuan Dairy Farm, Yangzhou University (Yangzhou, China). All procedures were conducted in the animal experiment were managed in accordance with the laws and regulations approved by the animal welfare office of Yangzhou University (YZUDWLL-201703-001).

There were 24 Holstein cows with similar body weights, milk yields, and parities used and divided into four groups with a randomized block design. All cows were housed in individual tie stalls and milked daily at 06:00, 14:00, and 20:00 h. All cows were cultured with a common total mixed ration (TMR) diet (Table 1) for 7 days, and then randomly assigned to four treatments: (i) Control (C)—cows were fed with a TMR diet only; (ii) T1—cows were fed with the TMR diet supplemented with 5 g/day of BM1259 powder (1 × 10^8^ cfu/g); (iii) T2—cows were fed with the TMR diet supplemented with 10 g/day of BM1259 powder (1 × 10^8^ cfu/g); and (iv) T3—cows were fed with the TMR diet supplemented with 15 g/day of BM1259 powder (1 × 10^8^ cfu/g). The experimental period was 11 weeks, consisting of a 2-week adaptation period and a 9-week sample collection period. All cows had free access to water throughout the entire experiment.

### 2.5. Sampling, Measurement, and Analyses

The offered and refused feed amounts were recorded on the third and fourth days every other week throughout the entire experimental period. All samples were dried in an oven at 65 °C for 48 h, ground with a Wiley mill to pass through a 2 mm screen, and then stored for further analyses.

Milk yield was recorded each day, and milk samples were collected on the last two days in each week in the experimental period. The 4% fat-corrected milk (FCM) yield was calculated using the following equation (1):

4% FCM (kg⁄day) = milk yield (kg⁄day) × (0.4 + 15 × fat content⁄100)(1)

Milk samples were composited proportionally (4:3:3, composite) for analyzing the contents [21]. A 50 mL subsample was treated with bronopol and stored at 4 °C for the determination of fat, true protein, and lactose contents (Shanghai Bright Holstein, Shanghai, China).

The rumen fluid samples were collected at 4 h after feeding on the morning of the last day of the experimental period. The rumen fluid was filtered through four layers of gauze, and then analyzed for pH immediately by a glass electrode pH meter. It was then centrifuged at 500× *g* for 5 min and the supernatants were stored at −20 °C for further analyses. Ruminal volatile fatty acids (VFA) were measured by gas chromatography (GC-14B; Shimadzu, Kyoto, Japan) equipped with a flame ionization detector.

Jugular blood samples of approximately 10 mL were collected from each cow in vacuum plasma tubes 3 h after the morning feeding on the third day of weeks 4, 8, and 12 [21]. The collected blood was then centrifuged at 3000× *g* at 4 °C for 10 min to collect serum, which was frozen at −20 °C for further analysis. Serum samples were analyzed for quantification of total protein (TP), albumin (ALB), globulin (GLOB), aspartate aminotransferase (AST), alanine aminotransferase (ALT), blood urea nitrogen (BUN), and uric acid (UA), according to methods described in previous studies [22].

### 2.6. Statistical Analysis

All data were analyzed using the MIXED procedure with repeated measurements with the SAS software system (SAS Institute Inc., Cary, NC, USA). Significance was declared at *p* < 0.05.

## 3. Results

### 3.1. Identification of BM1259

Bacterial cell morphology was observed using a scanning electron microscope. BM1259 showed rod-shaped cells that occurred in pairs or short chains (Figure 1B), with a cell length of up to 4.5 µm. Multiple alignments of 16S rRNA sequences revealed that BM1259 was approximately 97% similar to *B. megaterium* ATCC 14581 (Figure 1C). Additionally, phylogenetic analysis demonstrated the phylogenetic association of BM1259 with other completely sequenced bacterial genomes. Figure 1D indicates that BM1259 formed a separate clade with *B. megaterium* NBRC 15308^T^.

### 3.2. Genome Architecture and General Features of BM1259

**Figure 2:**
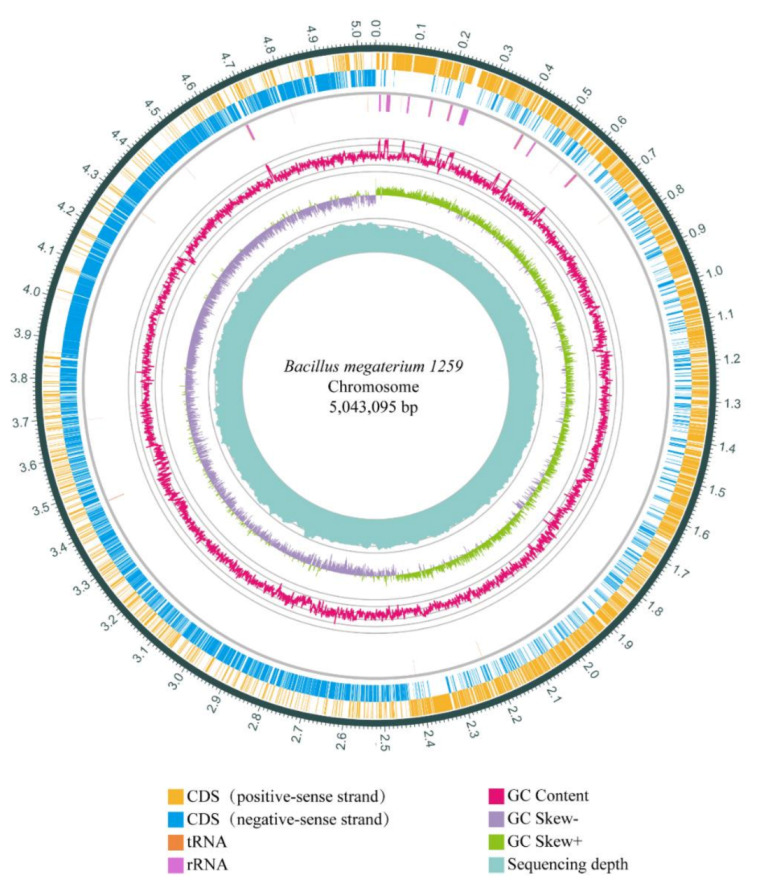
Circular chromosome of BM1259 genome. The first two outer circles indicate positive-sense and negative-sense strands with putative genes. The third circle represents tRNA and rRNA, depicted by orange and purple. The fourth and fifth circles indicate the GC content and the GC skew. The innermost circle represents the sequencing depth.

To investigate the genetic information of BM1259, we sequenced its entire genome. The genome of BM1259 contains a circular chromosome of 5,043,095 bp and three circular plasmids, BM1259 PM1 (159,518 bp), PM2 (72,779 bp), and PM3 (50,526 bp). The GC content of the chromosome is 38.25%. Local GC content variation and a clear-cut GC skew at the origin of replication (ori) are evident (Figure 2). A total of 5252 genes were analyzed from the chromosome of BM1259, which comprise 5092 coding genes (CDSs), 120 tRNAs, and 40 rRNAs (Table S1). Similar to the GC content, most genes are substantially distributed on the two DNA strands around the ori. In addition, most tRNA sequences are clustered and distributed close to the rRNA locus (Figure 2).

As a critical part of genome analysis for many other *B. megaterium* strains, the three largest plasmids of BM1259 were also analyzed and predicted in our study (Figure S1). The GC contents were 33.39%, 35.56%, and 34.00% for PM1, PM2, and PM3, respectively (Table S1). PM1 comprises 179 genes, consisting of 178 CDS and 1 tRNA (Table S1). In addition, PM2 comprises 73 protein-coding sequences, and PM3 expresses 36 CDSs, 18 tRNAs, and 3 rRNAs (Table S1). The plasmids contain 5.34% of the total gene number of BM1259.

The genome was screened for the presence of CRISPR elements and genomic islands. However, CRISPR elements and genomic islands were not found in the genome of BM1259.

### 3.3. Functional Annotation of Predicted Genes from the BM1259 Genome

After predicting the genes from the genome, all identified genes (100% coverage) were annotated based on a set of sequential BLAST searches using the GO, KEGG, COG, RefSeq, Pfam, and TIGRFAM databases (Table S2). COG analysis classified 3265 genes out of 5379 CDSs, which were divided into 26 categories (Table S2). The most coding genes were grouped into three main categories: transcription, amino acid transport, and metabolism and general function prediction only (Figure S2A). The pathway analysis of these differentially expressed proteins showed that 2534 new genes were categorized into five functional groups with a KEGG classification (Table S2). Among these KEGG categories, the cluster of “metabolism” occupied the highest number, of which, amino acid metabolism, carbohydrate metabolism, and metabolism of cofactors and vitamins were the main groups (Figure S2B). Notably, no pathogenic genes were found in our study, suggesting that BM1259 should be safe to use in feed additives.

### 3.4. Nitrate Assimilation-Related Genes and Pathway Identification

**Figure 3:**
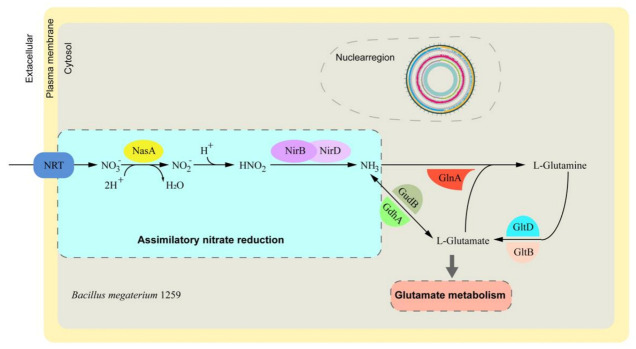
Model of the nitrate assimilation pathway in BM1259.

Depending on functional annotation, the genes involved in nitrate metabolism were screened and compared by performing BLASTP against the KEGG database. There were 12 nitrate assimilation-related genes identified from the annotated genes (Table S3). Among the 12 candidates, 9 genes of interest were found to be located in the nitrate assimilation pathway (Figure 3), indicating a potential role of BM1259 in nitrate metabolism in animals.

### 3.5. Effects of Dietary BM1259 on Milk Yield and Composition in Lactating Dairy Cows

To explore its effects on performance in lactating dairy cows, BM1259 was supplemented in the dairy cows’ diets. Although milk yield was not different among treatments, the 4% FCM yield of T3 was significantly higher than those of C and T1 (*p* < 0.05) but not that of T2 (Table 2). Except for milk urea nitrogen (MUN), other milk composition indicators (protein, fat, and lactose) were not affected by the addition of BM1259 to the diet. No significant difference was observed among treatments in TS and feed efficiency (Table 2). These results show that BM1259 could increase the 4% FCM production of Holstein dairy cows, although milk production was not affected by BM1259.

### 3.6. Effects of Dietary BM1259 on Ruminal Fermentation and Excrement Nitrogen Biochemical Indicators in Lactating Dairy Cows

The composition of propionate (C3) was higher in T2 than in the other treatments. In addition, T3 had a higher C2:C3 ratio than T2. No significant differences were found among the treatments in the ruminal pH, NH3-N content, total VFA concentration, or butyrate content (nC4) (Table 3). However, one of the novel findings in the present study is that BM1259 decreased the concentration of NH3-N in the feces (Table 4). The results of our study indicate that supplementation with BM1259 in the diet could enhance the nitrogen utilization efficiency and reduce the excretion of protein in the feces.

### 3.7. Effects of Dietary BM1259 on Blood Metabolites in Lactating Dairy Cows

T1 and T2 had significantly higher TP and GLOB concentrations than C. No differences were observed among treatments in ALB, AST, or UA concentrations (Table 5). The concentrations of ALT and BUN in T3 were higher than those in C (Table 5). In this study, blood metabolites (TP, ALB, GLB, AST, ALT, BUN, and UA) were within the normal ranges reported in the literature, which suggested that BM1259 had no negative effect on blood indicators.

## 4. Discussion

*B. megaterium*, named for its large size (approximately 1.5 × 5 µm), was first discovered by Anton De Bary in 1884 [23]. The newly identified BM1259 has similar morphological characteristics to other *B. megaterium* strains. In recent decades, several features of *B. megaterium* have been discovered, including sporulation, germination, antibiotic resistance, UV sensitivity [24,25,26], and production of substances [11,27,28], and a number of strains have been extensively studied genetically [27]. Two important *B. megaterium* strains, QM B1551 and DSM319, harbor 5,097,129 bp and 5,097,447 bp circular chromosomes, respectively. DSM319 is a natural strain with few plasmids, while QM B1551 contains seven indigenous plasmids, with sizes from 5.4 kb to over 164 kb, transcribing 8 to 176 genes [29]. Our genomic analysis showed that the circular chromosome of BM1259 was 5,043,095 bp in length, slightly smaller than those of QM B1551 and DSM319. BM1259 contains three circular plasmids, less than QM B1551 and more than DSM319. BM1259 possesses 5252 genes, which is similar to the number of QM B1551 or DSM319 genes. The genomic comparison showed the similarities and differences between BM1259 and other *B. megaterium* strains.

Bacteria utilize nitrate as an important source of nitrogen. Bioinformatics analyses of bacterial genomes have revealed structural and regulatory genes responsible for the nitrate assimilation phenotype [30]. Zhou’s team also found that *B. megaterium* NCT-2 can utilize nitrate as its only nitrogen source for growth and enhance nitrate removal from the soil [31,32]. Our analysis showed that BM1259 harbors the entire nitrate assimilation pathway, suggesting a potential role of BM1259 in nitrate metabolism in the animal gut environment.

Many reports have demonstrated that probiotics can improve the production of various animals [33,34,35,36]. Studies in chickens have shown that *Bacillus subtilis* can be used as a biocontrol agent to prevent pathogenic microorganisms [37,38] while also increasing digestive enzyme activity and reducing ammonia production [39], thereby improving the growth performance of poultry. Sun et al. reported that oral administration of *Bacillus subtilis natto* before weaning could reduce milking time and positively improve calf growth performance [40]. In our study, 4% FCM production was increased by adding BM1259 to the lactating dairy cow diet (Table 2).

Rumen fermentation and the stability of the intraruminal milieu are often reflected by the key ruminal indicators: ruminal pH, NH_3_-N, and total VFA. Ruminal pH was not affected by treatment in our study, which indicated that BM1259 did not have a negative effect on ruminal pH in lactating dairy cows (Table 3). The ruminal VFA concentration is associated with nutrient digestibility and pH [41,42]. Qiao et al. reported that higher VFA and acetate concentrations occurred when using *B. licheniformis* supplementation in Chinese Holstein cows [8]. In our study, there were no differences in total VFA concentration among treatments. Ruminal ammonia reduction may usually be caused by an increase in ammonia assimilation and microbial protein synthesis [43,44]. No difference was observed in ruminal ammonia production among treatments. However, supplementation with BM1259 caused a decrease in the concentration of NH_3_-N in the feces (Table 4), indicating that BM1259 has the potential to increase the nitrogen utilization efficiency in dairy cows. Song et al. [5] demonstrated that the addition of *B. subtilis natto* product to the diet did not affect the concentration of total dietary nitrogen but reduced the resultant concentration of fecal NH_3_-N, which is consistent with the results of our study. Fecal nitrogen is mainly composed of metabolic fecal nitrogen and undigested feed protein [45]. Previous studies showed that feeding layer hens [46] and broilers fermented products of *B. subtilis* significantly reduced the release of NH_3_-N in excreta [47]. The reduction in NH_3_-N in excreta implies that the addition of several *Bacillus* spp. can promote nitrogen utilization in livestock and poultry.

## 5. Conclusions

In summary, the addition of BM1259 to the diets of dairy cows in the lactation period had a positive effect on 4% FCM production. Moreover, supplementation with BM1259 increased C3 in the rumen and reduced NH_3_-N in the feces, indicating a possible improvement in nitrogen utilization. Our results have demonstrated that BM1259 (1 × 10^8^ cfu/g) may be a potentially useful probiotic in dairy cows, with a recommended dose of 10 or 15 g/day per head. Further research is necessary to uncover the mechanism by which BM1259 improves nitrogen utilization in lactating dairy cows.

## Figures and Tables

**Table 1:** Ingredients and chemical composition of the basal diet (%, as-fed DM).

Item	Value
Ingredients	
Ground corn	15.83
Flaked corn	5.72
Soybean meal	3.52
Cottonseed meal	2.86
Corn germ meal	2.5
Beet pulp	4
DDGS	5.68
Brewer grains	2.46
Soybean hull	5.8
Cottonseed	3
CaCO_3_	0.35
CaHPO_4_	0.44
NaCl	0.22
Sodium bicarbonate	0.33
Yeast culture	0.11
Alfalfa	4
Corn silage	43
Premix ^1^	0.18
Total	100
Nutrient levels	
CP	18.37
NDF	37.06
ADF	16.21
Ca	0.71
P	0.43
NE_L_/(MJ·Kg)	6.91

Note: DM, dry matter; CP—crude protein; NDF—neutral detergent fiber; ADF—acid detergent fiber; NE_L_—net energy for lactation. ^1^ Premix provided VA 3 000 IU, VD_3_ 1,400 IU, VE 30 IU, Fe 100 mg, Cu 10 mg, Zn 35 mg, Mn 20 mg, I 0.3 mg, Se 0.1 mg, Co 0.08 mg in the diet.

**Table 2:** Effects of dietary BM1259 on milk yield and composition in lactating dairy cows.

Items	C	T1	T2	T3
Yield, kg/d				
Milk	30.21 ± 2.40	32.42 ± 3.02	29.27 ± 4.31	30.49 ± 5.01
4% FCM	35.19 ± 3.84 ^a^	35.84 ± 2.07 ^a^	37.38 ± 0.56 ^ab^	39.25 ± 1.46 ^b^
Milk composition, %				
Protein	2.94 ± 0.26	2.99 ± 0.20	3.15 ± 0.24	3.11 ± 0.27
Fat	2.89 ± 0.26	3.12 ± 0.35	3.06 ± 0.27	3.09 ± 0.18
Lactose	5.06 ± 0.16	4.90 ± 0.04	4.54 ± 0.17	4.91 ± 0.11
TS	11.53 ± 2.05	11.63 ± 1.28	11.42 ± 0.95	11.74 ± 1.44
MUN	7.50 ± 0.42 ^a^	10.43 ± 0.36 ^b^	8.91 ± 0.22 ^ab^	10.24 ± 0.32 ^b^
Feed efficiency	1.34 ± 0.17	1.51 ± 0.43	1.81 ± 0.58	1.43 ± 0.34

Note: TS—total solid; MUN—milk urea nitrogen. C—fed with TMR diets only; T1—fed with TMR diet supplemented with 5 g/day of BM1259; T2—fed with TMR diet supplemented with 10 g/day of BM1259; T3—fed with TMR diet supplemented with 15 g/day of BM1259. ^a, b^ Different superscripts mark significant differences between the treatments (*p* < 0.05).

**Table 3:** Effects of dietary BM1259 on ruminal fermentation in lactating dairy cows.

Items	C	T1	T2	T3
Ruminal pH	6.42 ± 0.25	6.41 ± 0.25	6.30 ± 0.24	6.46 ± 0.25
NH_3_-N (mg N/dL)	17.40 ± 5.40	21.42 ± 2.26	20.95 ± 2.07	20.42 ± 5.19
Total VFA (mmol/L)	84.68 ± 6.06	92.52 ± 7.45	94.54 ± 8.40	93.02 ± 27.81
Acetate (C2) (%)	65.40 ± 0.01 ^a^	64.60 ± 0.02 ^a^	61.25 ± 0.04 ^b^	66.20 ± 0.01 ^a^
Propionate (C3) (%)	17.50 ± 0.01 ^a^	17.80 ± 0.01 ^a^	21.50 ± 0.05 ^b^	17.40 ± 0.01 ^a^
Butyrate (C4) (%)	13.40 ± 0.01	14.00 ± 0.01	12.50 ± 0.02	13.00 ± 0.01
C2:C3	3.73 ± 0.17 ^ab^	3.64 ± 0.27 ^ab^	3.35 ± 0.51 ^a^	3.84 ± 0.27 ^b^

Note: C—fed with TMR diets only; T1—fed with TMR diet supplemented with 5 g/day of BM1259; T2—fed with TMR diet supplemented with 10 g/day of BM1259; T3—fed with TMR diet supplemented with 15 g/day of BM1259. ^a,b^ Different superscripts mark significant differences between the treatments (*p* < 0.05).

**Table 4:** Effects of dietary BM1259 on nitrogen biochemical indicator of excrement in lactating dairy cows.

Items	C	T1	T2	T3
Feces				
BUN (mg/g)	4.36 ± 0.21 ^a^	3.98 ± 0.37 ^ab^	4.23 ± 0.26 ^ab^	4.01 ± 0.31 ^b^
UA (mg//g)	0.97 ± 0.03	1.07 ± 0.13	0.63 ± 0.09	0.72 ± 0.43
NH_3_-N (umol/g)	603.39 ± 91.22 ^a^	611.81 ± 87.36 ^ab^	573.53 ± 72.99 ^b^	581.00 ± 100.03 ^ab^
Urase (umol/g.24 h)	4.27 ± 0.76	4.36 ± 0.43	4.11 ± 0.58	4.56 ± 0.91
Uricase (%)	0.44 ± 0.01	0.31 ± 0.08	0.33 ± 0.07	0.49 ± 0.03
Urine				
BUN (mmol/L)	397.03 ± 50.91 ^a^	376.33 ± 71.23 ^ab^	346.33 ± 107.06 ^ab^	372.11 ± 61.23 ^b^
UA (mmol/L)	7.36 ± 0.91	8.22 ± 1.02	6.27 ± 2.11	6.11 ± 3.87

Note: BUN—blood urea nitrogen; UA—uric acid. C—fed with TMR diets only; T1—fed with TMR diet supplemented with 5 g/day of BM1259; T2—fed with TMR diet supplemented with 10 g/day of BM1259; T3,—fed with TMR diet supplemented with 15 g/day of BM1259. ^ab^ Different superscripts mark significant differences between the treatments (*p* < 0.05).

**Table 5:** Effects of dietary BM1259 on blood metabolites in lactating dairy cows.

Items	C	T1	T2	T3
TP (g/L)	77.18 ± 5.57 ^a^	85.56 ± 6.09 ^b^	88.28 ± 5.96 ^b^	83.19 ± 2.94 ^ab^
ALB (g/L)	29.77 ± 2.38	28.93 ± 1.77	27.49 ± 1.07	28.57 ± 1.01
GLOB (g/L)	45.04 ± 4.52 ^a^	60.15 ± 7.11 ^b^	60.09 ± 5.57 ^b^	55.70 ± 3.00 ^b^
AST (U/L)	68.27 ± 1.89	77.88 ± 4.45	82.56 ± 1.76	81.31 ± 1.25
ALT (U/L)	28.99 ± 12.37 ^a^	29.17 ± 8.67 ^a^	28.65 ± 9.40 ^a^	34.94 ± 3.60 ^b^
BUN (mmol/L)	4.30 ± 0.31 ^a^	4.80 ± 0.23 ^b^	4.60 ± 0.22 ^ab^	4.93 ± 0.35 ^b^
UA (µmol/L)	76.04 ± 7.50	80.33 ± 8.55	75.08 ± 2.33	77.55 ± 4.47

>Note: TP—total protein; ALB—albumin; GLOB—globulin; AST—aspartate aminotransferase; ALT—alanine aminotransferase; BUN—blood urea nitrogen; UA—uric acid. C—fed with TMR diets only; T1—fed with TMR diet supplemented with 5 g/day of BM1259; T2—fed with TMR diet supplemented with 10 g/day of BM1259; T3—fed with TMR diet supplemented with 15 g/day of BM1259. ^ab^ Different superscripts mark significant differences between the treatments (*p* < 0.05).

## Data Availability

No new data were created or analyzed in this study. Data sharing is not applicable to this article.

## Supplementary Materials

The following are available online at https://www.mdpi.com/2076-2615/11/2/397/s1, Figure S1: Circular maps of BM1259 plasmids PM1, PM2 and PM3, Figure S2: Function Classification of annotated genes from BM1259 genome, Table S1: The Genome features of BM1259, Table S2: The summary statistics for functional annotation of genes from BM1259, Table S3: The genes of BM1259 associated with nitrogen metabolism.
