# Supplementary material for: Genomics Analysis of Bacillus megaterium 1259 as a Probiotic and Its Effects on Performance in Lactating Dairy Cows
Source: Animals (Basel). 2021 Feb 4;11(2):397. doi: 10.3390/ani11020397 (PMC7914491; doi:10.3390/ani11020397)
Supplement: Supplementary file 1 [file animals-11-00397-s001.pdf]

Supplementary Material

Figure S1. Circular maps of BM1259 plasmids PM1, PM2 and PM3. The first two outer circles indicate positive-sense and negative-sense strand with putative CDS, tRNA and rRNA depicted by blue, orange and pink respectively. The black and green/purple circles indicate GC content and GC skew.

Figure S2. Function Classification of annotated genes from BM1259 genome. (A) COG categories of annotated genes; (B) KEGG categories of annotated genes.

Table S1. The Genome features of BM1259.

Table S2. The summary statistics for functional annotation of genes from BM1259.

Table S3. The genes of BM1259 associated with nitrogen metabolism.

Figure S1.

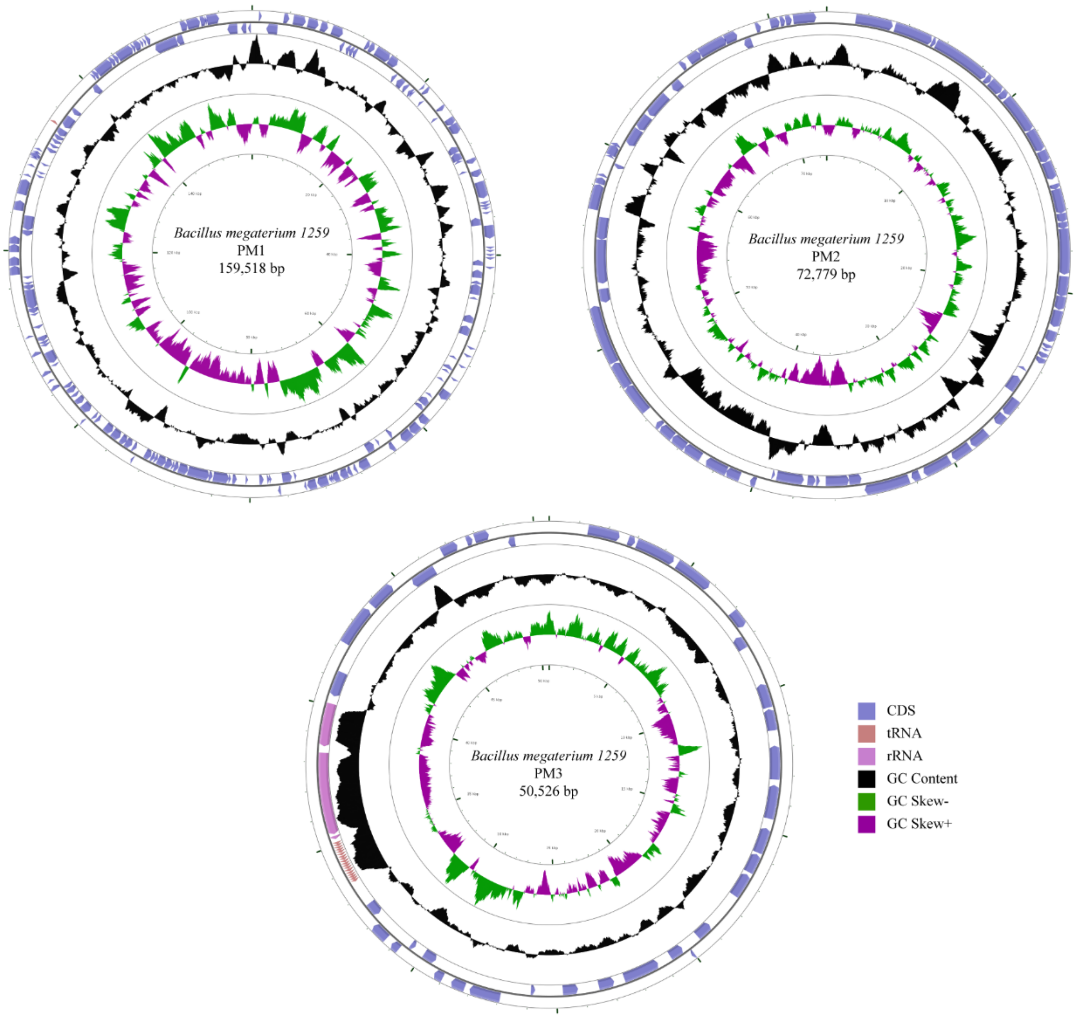
Bacillus megaterium 1259
PM1
159,518 bp
Bacillus megaterium 1259
PM2
72,779 bp
Bacillus megaterium 1259
PM3
50,526 bp
CDS
tRNA
rRNA
GC Content
GC Skew-
GC Skew+

Figure S2.

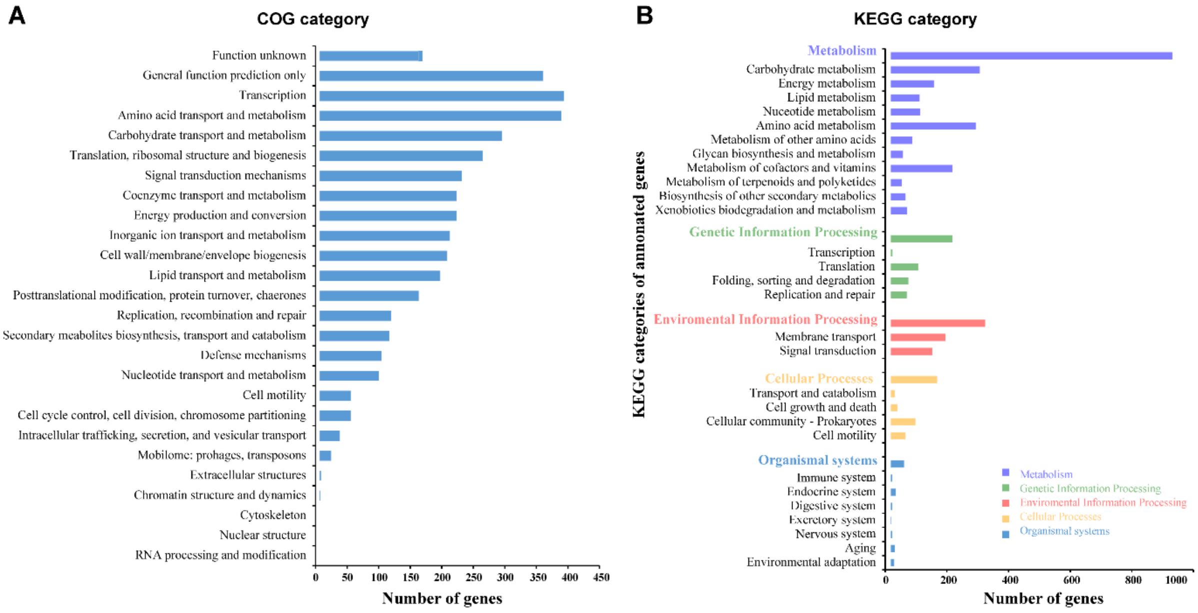

A
COG category
Function unknown
General function prediction only
Transcription
Amino acid transport and metabolism
Carbohydrate transport and metabolism
Translation, ribosomal structure and biogenesis
Signal transduction mechanisms
Coenzyme transport and metabolism
Energy production and conversion
Inorganic ion transport and metabolism
Cell wall/membrane/envelope biogenesis
Lipid transport and metabolism
Posttranslational modification, protein turnover, chaerones
Replication, recombination and repair
Secondary metabolites biosynthesis, transport and catabolism
Defense mechanisms
Nucleotide transport and metabolism
Cell motility
Cell cycle control, cell division, chromosome partitioning
Intracellular trafficking, secretion, and vesicular transport
Mobilome: prohages, transposons
Extracellular structures
Chromatin structure and dynamics
Cytoskeleton
Nuclear structure
RNA processing and modification
0 50 100 150 200 250 300 350 400 450
Number of genes
B
KEGG category
KEGG categories of annonated genes
Metabolism
Carbohydrate metabolism
Energy metabolism
Lipid metabolism
Nuceotide metabolism
Amino acid metabolism
Metabolism of other amino acids
Glycan biosynthesis and metabolism
Metabolism of cofactors and vitamins
Metabolism of terpenoids and polyketides
Biosynthesis of other secondary metabolites
Xenobiotics biodegradation and metabolism
Genetic Information Processing
Transcription
Translation
Folding, sorting and degradation
Replication and repair
Enviromental Information Processing
Membrane transport
Signal transduction
Cellular Processes
Transport and catabolism
Cell growth and death
Cellular community - Prokaryotes
Cell motility
Organismal systems
Immune system
Endocrine system
Digestive system
Excretory system
Nervous system
Aging
Environmental adaptation
Metabolism
Genetic Information Processing
Enviromental Information Processing
Cellular Processes
Organismal systems
0 200 400 600 800 1000
Number of genes

Table S1. The Genome features of BM1259.

	Size (pb)	CDS	tRNA	rRNA	GC content (%)	Topology	Sequencing depth (X)
Chromosome	5,043,095	5092	120	40	38.25	circular	68.04
PM1	159,518	178	1	0	33.39	circular	61.34
PM2	72,779	73	0	0	35.56	circular	40.35
PM3	50,526	36	18	3	34.00	circular	103.89

Table S2. The summary statistics for functional annotation of genes from BM1259.

Database	Annotated number	% all
COG	3265	60.70
KEGG	2534	47.11
GO	2879	53.52
Refseq	5269	97.96
Pfam	4396	81.73
TIGRFAMs	1456	27.07
All databases	1045	19.00
At least one databases	5271	97.00
Overall	5379	100

Table S3. The genes of BM1259 associated with nitrogen metabolism.

Gene name	Gene ID	Function description	BlastX best hit	Protein length (aa)	E-value
NirB	ko:K00362	nitrite reductase (NADH) large subunit	bmq:BMQ_0777	804	0
NasA	ko:K00372	assimilatory nitrate reductase catalytic subunit	bmq:BMQ_0778	716	0
NRT/Nark	ko:K02575	MFS transporter, NNP family, nitrate/nitrite transporter	bmq:BMQ_0779	393	0
NirD	ko:K00363	nitrite reductase (NADH) small subunit	bmq:BMQ_1154	108	5.3E-74
GdhA	ko:K00262	glutamate dehydrogenase (NADP+)	bmeg:BG04_4022	460	0
GltB	ko:K00265	glutamate synthase (NADPH/NADH) large chain	bmh:BMWSH_3162	1517	0
GltD	ko:K00266	glutamate synthase (NADPH/NADH) small chain	bmh:BMWSH_3161	493	0
GudB	ko:K00260	glutamate dehydrogenase	bmd:BMD_2413	428	0
Can/CynT	ko:K01673	carbonic anhydrase	bmq:BMQ_2597	195	9.89E-143
GlnA	ko:K01915	glutamine synthetase	bmh:BMWSH_1129	444	0
NarQ	ko:K07674	two-component system, NarL family, nitrate/nitrite sensor histidine kinase NarQ	bmh:BMWSH_1936	273	0
NirC	ko:K02598	nitrite transporter	bmeg:BG04_3458	278	0
